# Malignant Ventricular Arrhythmia With Apical Biventricular Noncompaction

**DOI:** 10.7759/cureus.73323

**Published:** 2024-11-09

**Authors:** Lakshya Seth, Vraj Patel, Khyati Pandya

**Affiliations:** 1 Internal Medicine, Augusta University Medical College of Georgia, Augusta, USA; 2 Pediatric Cardiology, Augusta University Medical College of Georgia, Augusta, USA

**Keywords:** arrhythmia, cardiology, echocardiogram, electrocardiogram, implantable cardioverter defibrillator, internal medicine, pediatric cardiology, pediatrics, ventricular fibrillation, ventricular tachycardia

## Abstract

Ventricular tachycardia (VT) arising from the right ventricle outflow tract is the most common cause of VT in children with a structurally normal heart. It often presents as a monomorphic VT that is usually amenable to ablation during an electrophysiology (EP) study. VT in children is typically idiopathic and benign but carries a risk for the degeneration of the arrhythmia into ventricular fibrillation or can result in sudden cardiac death. We present a unique case of a patient with a history of recurrent palpitations and chest tightness who was found to have a significant burden of malignant ventricular arrhythmias in the presence of apical biventricular noncompaction and preserved ventricular systolic function. The patient was referred for an EP study to assess for ablation if a discrete focus could be identified. During the EP study, the arrhythmia degenerated into ventricular fibrillation that required prompt defibrillation with a manual defibrillator and placement of a single-chamber transvenous implantable cardioverter defibrillator (ICD). This case report highlights how physicians should be mindful that although most cases of VT in children are idiopathic and benign, there is a risk of degeneration of the arrhythmia into ventricular fibrillation or resulting in sudden cardiac death. Prompt recognition of concerning VT with the involvement of EP in the patient’s care is important.

## Introduction

Ventricular tachycardia (VT) is defined as an arrhythmia with three or more consecutive beats arising from the ventricle at a rate of more than 100 beats per minute [1,2]. Monomorphic VT is defined as VT with a single QRS morphology and a regular rate [2,3]. VT is uncommon in children, and in contrast to adults, in whom VT is associated with underlying ischemic heart disease, VT in children is most commonly idiopathic [4,5], with the right ventricular outflow tract being the most common origin of VT in children [6-8]. Though uncommon in children, VT carries a risk for degeneration into ventricular fibrillation [9] and sudden cardiac death [5,7,9].

Left ventricular noncompaction (LVNC) is a congenital heart defect thought to arise from abnormal myocardial compaction and characterized by prominent trabeculae and intertrabecular recesses, which result in a thickened myocardium [10,11]. These changes are most prominently noted in the left ventricular apex [12,13]. LVNC has been documented as a structural cause of VT in children [6,14], and a study conducted at Texas Children’s Hospital found that VT was the most common arrhythmia found in children with LVNC, accounting for over half of the cases [15].

We report a case of monomorphic VT in a pediatric patient in the background of biventricular noncompaction, which led to inducible ventricular fibrillation during an electrophysiology (EP) study that required defibrillation and placement of a transvenous implantable cardioverter defibrillator (ICD).

## Case presentation

**Figure 1:**
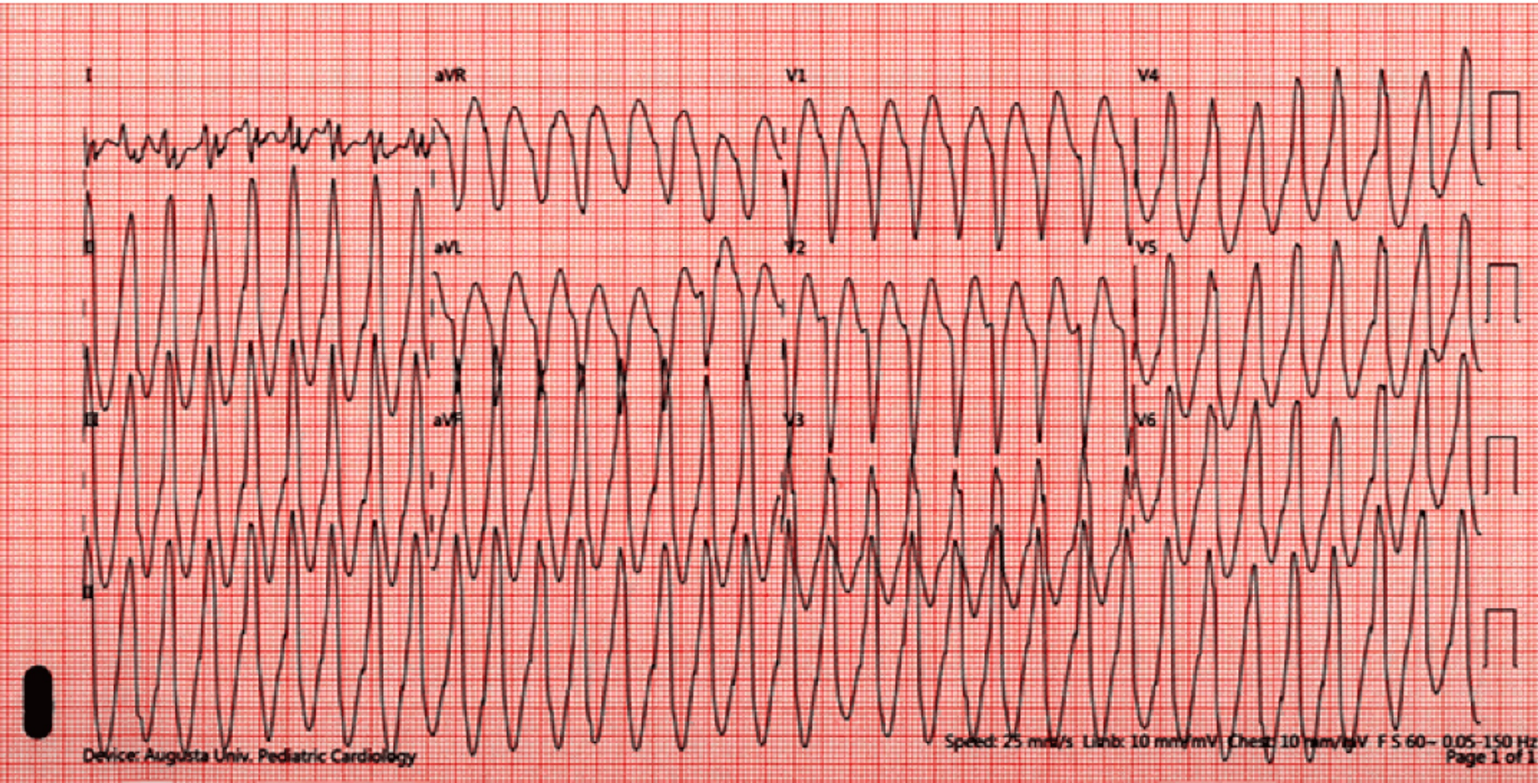
A stress test demonstrating recurrent episodes of monomorphic ventricular tachycardia.

A 15-year-old otherwise healthy girl presented with a history of recurrent palpitations and associated chest tightness for one year. Episodes lasted for several minutes at a time and were not associated with exertion. An episode recorded on a smartwatch showed premature ventricular contractions (PVCs) in a bigeminal pattern. She was evaluated with a stress test to look for worsening PVCs with exercise, an echocardiogram to evaluate cardiac anatomy, a cardiac MRI to rule out myocardial scarring or structural heart disease (SHD), a seven-day Holter monitor to assess for PVC burden and correlation with symptoms, and a genetic panel to assess for cardiac channelopathies. A stress test revealed recurrent episodes of monomorphic VT with a maximum rate of 148 bpm (Figure 1). The vital sign at rest is a blood pressure of 107/68, with a heart rate of 105. The vital sign during the stress test is 137/75, with a maximum heart rate of 148.

**Figure 2:**
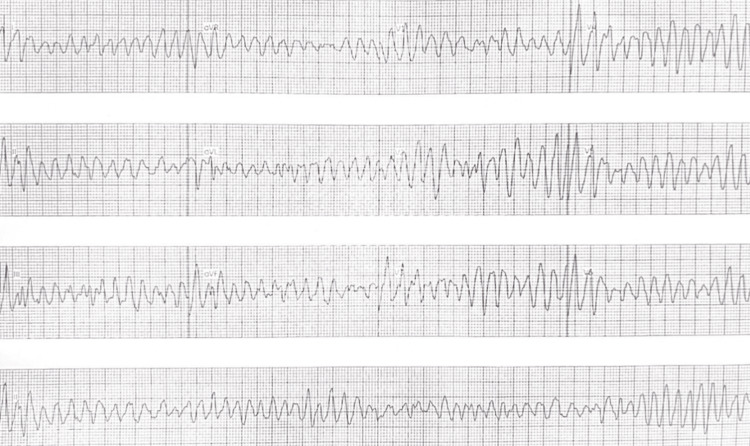
An intraoperative EKG reading demonstrating deterioration of the rhythm into ventricular fibrillation after administration of a stimulus from the right ventricular apex.

An echocardiogram revealed a structurally normal heart with normal proximal origins of the coronary arteries. The ventricular dimensions were within normal limits. The right ventricular systolic pressure was within normal limits based on the velocity of the tricuspid regurgitation jet. No other hemodynamic data was available as the patient did not undergo a cardiac catheterization. Cardiac MRI revealed noncompaction in the left and right ventricles that was not previously seen on the echocardiogram. Holter monitoring showed 1,252 episodes of sustained VT with a maximum heart rate of 234 bpm and a PVC burden of 26%. The arrhythmia panel was negative for heritable cardiac arrhythmias. The patient was started on 50 mg of atenolol daily due to the ease of dosing, which resulted in almost complete suppression of the VT. A repeat stress test after initiating atenolol showed no episodes of VT and no significant ST-T changes. The vital sign at rest was a blood pressure of 132/55, with a heart rate of 43. The vital sign during the stress test was 161/71, with a maximum heart rate of 124. She was subsequently referred for an EP study to assess for mapping and potential ablation of PVCs if a discrete focus could be identified. During the EP study, a triple ventricular stimulus from the right ventricular apex induced a sustained episode of polymorphic VT, which quickly degenerated into ventricular fibrillation (Figure 2).

**Figure 3:**
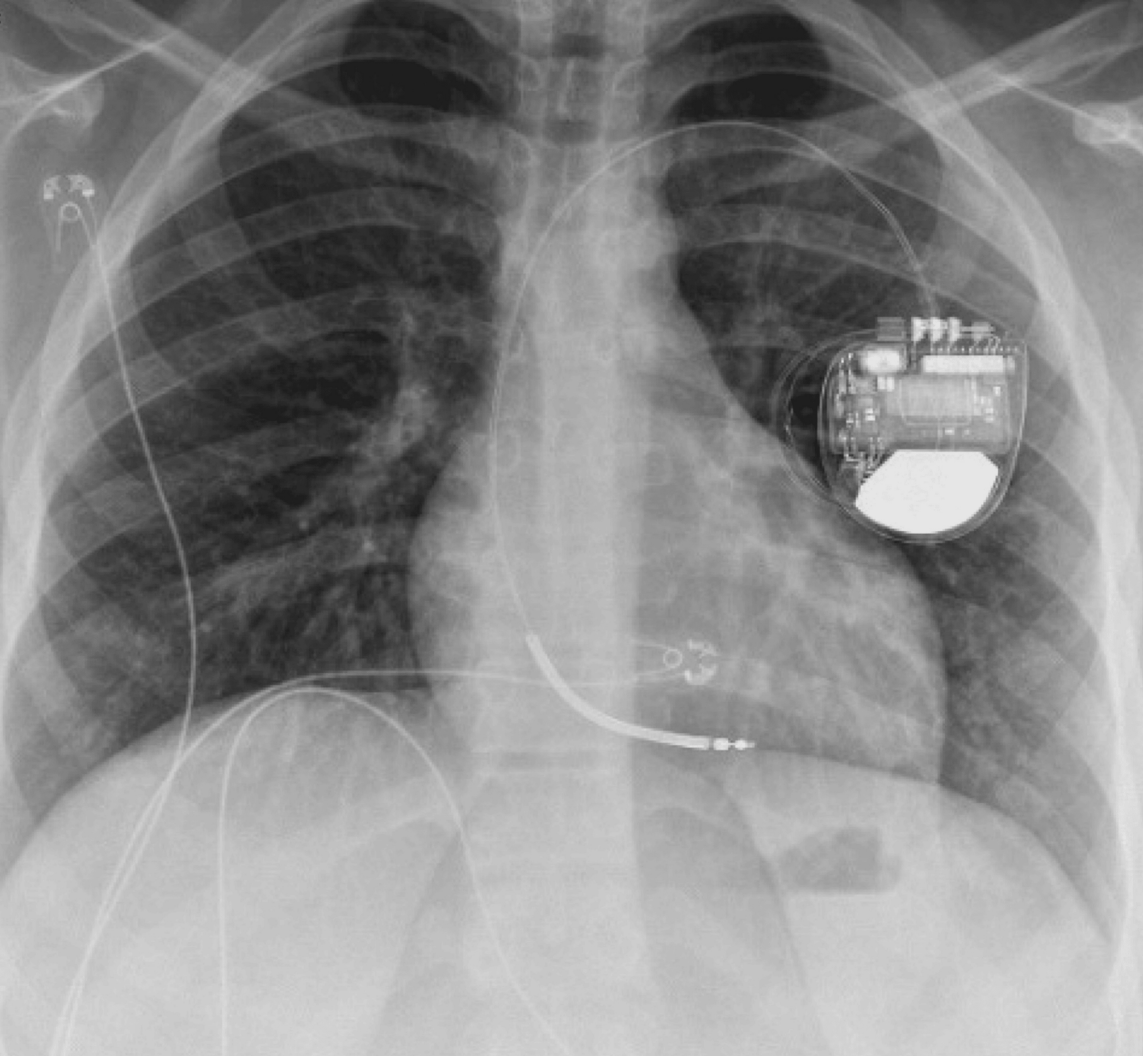
A chest X-ray confirming successful implantation of the single-chamber transvenous implantable cardioverter defibrillator.

Prompt defibrillation was performed with 200 J via a manual defibrillator, and the patient reverted to sinus rhythm. At this point, the EP study was terminated, and the patient was prepped for placement of a single-chamber transvenous ICD (Figure 3). Defibrillation threshold (DFT) testing was successfully performed after placement of the ICD. The patient was discharged home after an overnight stay in the pediatric intensive care unit (ICU).

## Discussion

VT is an arrhythmia that can occur in children with both structurally normal and abnormal hearts [16]. Characteristics of benign VT include a 24-hour PVC burden of less than 20%, no left ventricle dysfunction, and no presence of complex features (bigeminy, multiform, couplets, or nonsustained VT) [16]. Characteristics of malignant VT include a 24-hour PVC burden of more than 20%, polymorphic VT, left ventricle dysfunction, and the presence of complex features [16]. This patient presented with three concerning features of potentially malignant VT: a PVC burden of 26%, bigeminy, and evidence of LVNC on cardiac MRI. Of note, ventricular fibrillation was not one of the arrhythmias found in the Texas Children’s Hospital study investigating children with LVNC [15], nor was it one of the arrhythmias recorded in studies that investigated potential arrhythmias in adults with LVNC [17-19].

Genetic testing for cardiac channelopathies, cardiomyopathies, and SHDs is warranted in at-risk individuals in whom a heritable etiology is considered likely. Special regard must be placed when considering genetic testing in a pediatric population in terms of the likelihood of disease manifestation during childhood, the appropriate age to conduct genetic testing, the availability of effective therapies if a genetic cause is confirmed, and family preference [20]. LVNC is a structural cause of VT in children, and genetic mutations have been found to contribute to the phenotypic variability in presentation, warranting genetic testing in this population as well [15]. Treatment for VT in children revolves around the use of antiarrhythmic drugs such as beta-blockers [4,5,9] with more invasive treatment options, such as catheter ablation or ICD placement, reserved for those who are persistently symptomatic despite medical management [5]. The prognosis is good for patients with monomorphic VT [5] and is more successfully controlled than in those with polymorphic VT [9]. Patients with nonsustained VT [4] or those without underlying SHD [9] may experience spontaneous resolution without treatment. The prognosis for polymorphic VT and those with underlying heart disease is poor and may require ICD implantation [4]. The mortality rate of sustained VT is higher in cases with underlying SHD and can be resistant to treatment [5]. Complications such as sudden death and cardiac arrest more commonly develop in patients with polymorphic or sustained VT [9].

## Conclusions

This is a unique case of a patient with a significant burden of malignant ventricular arrhythmias in the presence of apical biventricular noncompaction and preserved ventricular systolic function. This case report highlights how physicians should be mindful that although most cases of VT in children are idiopathic and benign, there is a risk of degeneration of the arrhythmia into ventricular fibrillation or resulting in sudden cardiac death. Prompt recognition of concerning VT with the involvement of EP in the patient’s care is important. This case provides an example of the workup required in a pediatric patient who presented with symptomatic VT with concerning features. Ultimately, an EP study was unsuccessful, and this patient required ICD implantation due to degeneration of the arrhythmia into ventricular fibrillation.
